# Genetic surveillance of endemic bovine *Salmonella *Infantis infection

**DOI:** 10.1186/1751-0147-49-15

**Published:** 2007-05-11

**Authors:** Nanna Lindqvist, Sinikka Pelkonen

**Affiliations:** 1 Kuopio Research Unit, Department of Animal Diseases and Food Safety Research, Evira, Finnish Food Safety Authority, Kuopio, Finland

## Abstract

**Background:**

*Salmonella *serovar Infantis is endemic in Finnish food-producing animals since the 1970s. The purpose of this study was to describe the molecular epidemiology of the infection in cattle during 1985–2005, to follow the persistence of the feed-related outbreak strain from 1995 in the cattle population, and to analyse the stability of *Xba*I-banding patterns in individual herds during long-lasting infections.

**Methods:**

*Salmonella *Infantis isolates from 478 cattle herds (n = 588), covering 73% of the subclinically or clinically infected herds, were typed by pulsed-field gel electrophoresis (PFGE) using *Xba*I. DNA fragments larger than 125 kb were counted in PFGE types because of high plasmid background. Ribotyping and IS*200*-typing with *Ban*I-digested DNA were done on 57 selected isolates.

**Results:**

The isolates associated with the infection consisted of 51 PFGE types with genetic similarity (F value) between 0.58 and 0.95. From 1985 to 2003, the major type appeared on 68% of the farms. The three most common types, with F values of 0.90 to 0.95, accounted for 80% of the isolates. Only 17% of the isolates had F values below 0.80, and 1% below 0.70. Ribotyping and IS*200*-typing classified 89% of the analysed isolates into the major ribotype and IS*200 *type combination, and the rest fell into closely related types. Analysis of successive isolates from 142 herds revealed changes in *Xba*I-banding patterns in 21% of the herds with two analysed isolates and in 38% of the herds from which three or more isolates were analysed. The feed-related *S*. Infantis genotype from the 1995 outbreak had disappeared by 1999, at the time when the incidence of bovine salmonella, and *S*. Infantis in particular, strongly decreased.

**Conclusion:**

The study showed how genetic surveillance, as part of salmonella control, provides tools to follow the persistence of particular infections, and to assess the efficacy of control measures. Testing of several isolates from a herd in outbreak investigations is advisable, because minor changes in PFGE banding patterns frequently occur during long-lasting infections.

## Background

*Salmonella enterica *subspecies *enterica *serovar Infantis (referred to as *S*. Infantis) belongs to the ten most common salmonella serovars in Europe [1]. It has been, along with *S*. Typhimurium, the major endemic salmonella serovar in production animals in Finland since the early 1970s, when it contaminated the broiler production chain [2]. With the exception of 2003, *S*. Infantis has been yearly isolated from chicken flocks since 1971, and it accounted for 60% of the salmonella infections in flocks during 1995–2004. The *S*. Infantis infection spread to cattle, and bovine infections have been detected annually in 1980–2003, and again in 2006 [3] [S Pelkonen, unpublished].

**Figure 1:**
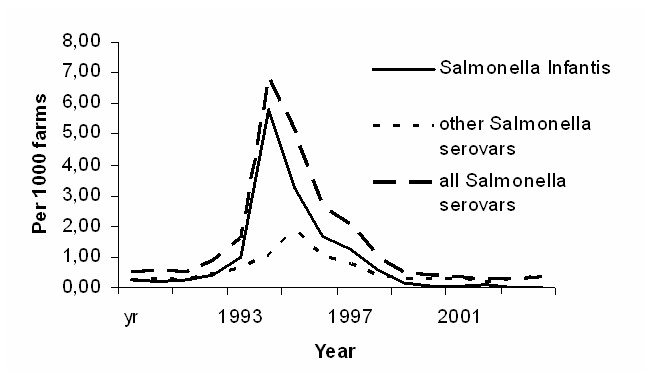
Annual incidence of farms (per 1000 farms) infected with *S*. Infantis or other *Salmonella *serovars in 1990–2005.

The share of *S*. Infantis-infected farms of the farms infected with all salmonella serovars ranged from 3 to 17% until 1985, rose to 19–30% in the late 1980s, and was at its highest (41–85%) in the 1990s. In the 2000s, the infection has almost disappeared. The highest incidence of *S*. Infantis positive cattle farms was connected to a domestic feedborne *S*. Infantis outbreak in 1995 [4]. Even then, only 0.578% of all cattle farms were infected with *S*. Infantis (Fig. 1).

Salmonella in general has extremely low prevalence in Finnish production animals. Systematic control of salmonella at the primary production stage has been practised in Finland for more than 40 years. Besides measures taken to combat salmonella infections in food-producing animals, the control has included monitoring of salmonella in feeding stuffs. The Finnish Salmonella Control Programme, approved by the European Commission Decision 94/968/EC, was launched in 1995. Its objective is to maintain the apparent salmonella prevalence below 1% in Finnish cattle and beef. The programme includes both surveillance at slaughterhouses and in herds, and sanitary and eradication measures if salmonella is detected from samples taken for monitoring or from a clinical case. Genetic typing of salmonella, as an auxiliary means of the programme, provides tools to follow the persistence of particular infections, to recognise new infections and to assess the efficacy of control measures.

Pulsed-field gel electrophoresis is a golden standard method for genetic differentiation of salmonella strains for epidemiological purposes (e.g. PulseNet, Enternet). Typically one isolate per herd is available for outbreak investigations, or stored in strain collections for epidemiological purposes, and results are based on this material. Literature on the genetic stability of salmonella strains in bovine infections is scant.

In the present investigation, we analysed the molecular genetic history of *S*. Infantis infection in Finnish cattle from 1985 to 2005, a time period from which isolates were available. We also analysed the stability of *Xba*I-banding patterns in individual herds during long-lasting infection. The aims of our study were to: 1) examine the genetic diversity among *S*. Infantis isolates from Finnish cattle during two decades, 2) follow the persistence of the feed-related outbreak strain from 1995 [4] in the cattle population, and 3) learn about genetic variation in *S*. Infantis isolates obtained from the same herd.

## Methods

### Bacterial isolates

**Figure 2:**
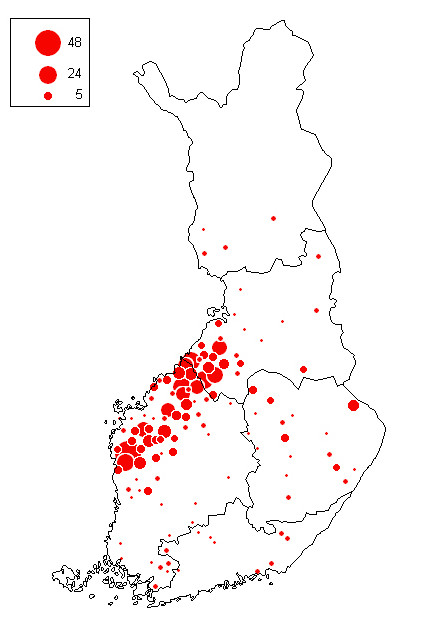
**Geographic distribution of *S*. Infantis cattle farms detected from 1993 to 2005**. Each *S*. Infantis positive farm (n = 478) is shown only once, although *S*. Infantis isolations were made on some farms in several years. The size of the circle indicates the number of farms (scale in figure) within a municipality.

*S*. Infantis isolates (total n = 588) were obtained from the laboratories of the former National Veterinary and Food Research Institute (current name Finnish Food Safety Authority Evira, Department of Animal Diseases and Food Safety Research) in Helsinki, Kuopio, Oulu, and Seinäjoki. The isolates originated from 478 cattle farms, which represented 73% of all 651 Infantis infected farms (Table 1) and their geographical distribution (Fig. 2). The collection included two or more isolates from 142 farms, taken during the eradication of the infection. Therefore, when the overall results were presented, generally only one isolate per farm per year was included (Table 2). However, if a farm had several profiles in the same year, all profiles (Table 2; n = 494) were included. All isolates had been serologically confirmed to be *S*. Infantis and stored at -70°C, except for the isolates from the 1980s, which prior to analysis were stored on egg agar slopes.

**Table 1:** *S*almonella Infantis in Finnish cattle from 1980 to 2005.

Year	No of analysed isolates	No of analysed farms	No of *S*. Infantis farms*	No of salmonella farms*
1980	0	0	1	32
1981	0	0	5	41
1982	0	0	8	51
1983	0	0	3	52
1984	0	0	6	67
1985	10	10	16	92
1986	13	12	29	110
1987	10	9	20	74
1988	0	0	14	75
1989	0	0	14	47
1990	0	0	14	28
1991	0	0	12	29
1992	4	4	12	26
1993	19	13	19	42
1994	28	26	45	75
1995	300	240	242	286
1996	144	106	125	196
1997	20	18	60	97
1998	22	22	43	69
1999	10	10	18	31
2000	3	3	5	14
2001	2	2	2	11
2002	1	1	1	9
2003	2	2	2	7
2004	0	0	0	7
2005	0	0	0	8

Total no.	588	478	651	1576

*obtained from [3]

Number of analysed *S*. Infantis isolates and farms, number of farms infected with *S*. Infantis and with any salmonella serovar.

**Table 2:** *Xba*I-PFGE types of *S*. Infantis isolates on 478 cattle farms.

	PFGE type*				
Year	type 1	type 2	type 3	other types **	Total
1985	9			1	10
1986	9	1		2	12
1987	5	1		3	9
1992	2	1		1	4
1993	11			3	14
1994	15	6		7	28
1995	179	12	11	41	243
1996	70	12	7	27	116
1997	11	2	2	3	18
1998	13	2	2	5	22
1999	7	2		1	10
2000	2			1	3
2001	1			1	2
2002				1	1
2003	1			1***	2

	335	39	22	98	494

*Only bands larger than 125 kb of the *Xba*I profile were regarded. Generally, one PFGE type per farm per year was included. If a farm had several types in the same year, all profiles were included.

**48 different PFGE types.

***A new type not seen previously. Resembles PFGE type 167 (Fig. 2), with two additional bands of 125 and 140 kb.

### Pulsed-field gel electrophoresis (PFGE)

PFGE using *Xba*I restriction enzyme and S1 nuclease was performed as described [4]. *Xba*I macrorestriction profiles differing by one or more DNA fragments larger than 125 kb were assigned a PFGE type number [4]. When discrimination of the *Xba*I-PFGE analysed isolates was based on all visible DNA fragments, regardless of their size and intensity, the macrorestriction profiles could be further divided into subtypes [4]. The coefficient of similarity (F) values between PFGE profiles were calculated as described [5]. PFGE patterns were also analysed using a computer program for analysis of electrophoretic patterns (GelCompar, Applied Maths, Kortrijk, Belgium) [6] to generate dendrograms.

### Ribotyping and IS*200*-typing

Ribotyping and IS*200*-typing were performed essentially as described [4,7] with *Ban*I-digested DNA; *EcoR*I digestion (New England Biolabs, Beverly, MA) was used to confirm some IS*200 *types [7]. The IS*200 *and 16S rRNA probes were labelled with DIG-11-dUTP by using a DIG-High Prime Labeling Kit (Boehringer Mannheim GmbH) and the analysis of *Salmonella *DNA completed as previously described [4]. Fiftyseven isolates were analysed by ribotyping and IS*200*-typing: 25 isolates from 1985 to 1987, 19 isolates from 1992 to 1995 and 13 isolates from 1999 to 2002.

## Results

### PFGE types and their subtypes

**Figure 3:**
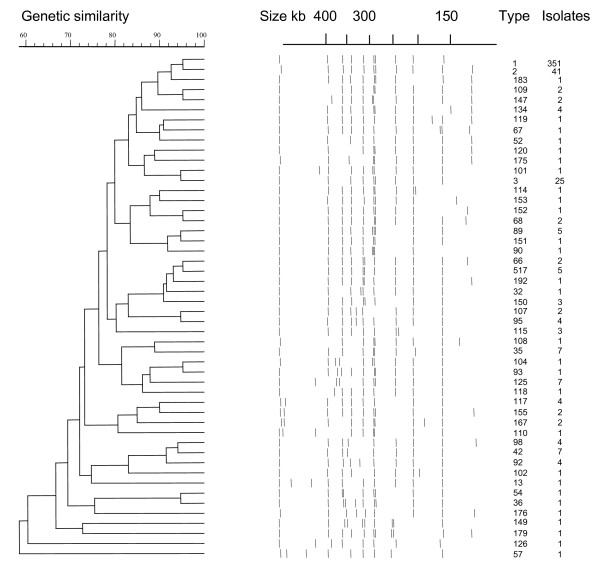
**Dendrogram of 50 PFGE types obtained by *Xba*I-PFGE of *S*. Infantis isolates**. Only bands larger than 125 kb were counted. The top bar on the left hand side indicates the similarity, with a value of 70 corresponding to an F value of 0.7. The top bar on the right hand side shows the molecular sizes in kilobases (kb). The numbers on the right hand side of the dendrogram and bands indicate the PFGE type (ranging from 1 to 517) and the number of isolates (ranging from 1 to 351) within each type.

Using *Xba*I we detected 51 PFGE types among 494 *S*. Infantis isolates from 478 farms (Table 2; Fig. 3), when DNA fragments larger than 125 kb were regarded. Bands corresponding to smaller fragments were excluded, because S1-nuclease analysis revealed plasmids in the size range of 20 to 125 kb in 88% of the analysed isolates (data not shown). Plasmids were more common in the isolates from 1995 to 2002 (313/332; 94%) than in the older isolates (39/66; 59%).

The predominant type 1 accounted for 68% (335/494) of the *S*. Infantis *Xba*I profiles from 478 farms detected in 1985 – 2005 (Table 2). The most common PFGE types 1, 2, and 3 accounted for 80% (396/494) of the profiles (Table 2). There was a one-band-difference between types 1 and 2 as well as between types 1 and 3; type 2 had an additional band of approximately 130 kb and type 3 lacked a band of approximately 350 kb compared to type1 (Fig. 3). Overall, the coefficient of similarity (F) values ranged from 0.58 to 0.95 (Fig. 3). The F values for the three most common types 1, 2 and 3, when compared to each other, were between 0.90 and 0.95. Thirteen PFGE types with altogether 409 isolates had F values between 0.83 and 0.95; that was 83% of the isolates. Only seven types, one isolate in each type, had F values below 0.7 (1% of the isolates). Nine types, and 25 isolates, had F values between 0.71 and 0.75 (5% of the isolates), and 21 types (52 isolates) had F values between 0.76 and 0.80 (11% of the isolates) (Fig. 3).

When all visible DNA fragments larger than 20 kb were regarded, PFGE types 1, 2, and 3 were subdivided into 41, 17 and 6 subtypes, respectively. The remaining 48 PFGE types were subdivided into 78 subtypes (data not shown).

### Occurrence of the feed-related PFGE type of 1995

The feedborne *S*. Infantis strain from 1995 belonged to PFGE type 1, carried a typical plasmid of 60 kb, and included the subtypes 1/39, 1/43, 1/44, 1/45 and 1/46 [4]. In the outbreak year 1995, the feed-related strains accounted for 36% of bovine *S*. Infantis infections [4]. In 1996, 12 farms had subtype 1/39, 2 farms type 1/43, 2 farms type 1/44, and 6 farms type 1/45. Feed-related type 1/45 was detected on 2 farms in 1997 and 1 farm in 1998. Of all *S*. Infantis infections on the analysed cattle farms (Table 1), the feed-related subtypes accounted for 19% (22/116) in 1996, 11% (2/18) in 1997 and 5% (1/22) in 1998. These subtypes were not detected in 1999 or 2000, but in 2001, one farm had 1/39 (data not shown).

### Regional clustering of PFGE types

Types 1 (with its subtypes 1/24, 1/34 and 1/37), 2 and 3 were common among cattle overall (Table 2; data not shown). However, there was clustering of types and their subtypes in certain municipalities at the time of the highest salmonella prevalence in 1995–1996: 1/24 was seen in Kälviä in all four analysed isolates, in Ilmajoki in 3 out of 6 (3/6) analysed isolates and in Vihanti in 3/8 analysed isolates. In Teuva, type 1/37 had spread (5/23 analysed isolates), in Kaustinen type 2 (6/13 analysed isolates) and in Himanka type 3 (3/4 analysed isolates). These municipalities are located in the western part of Finland (Fig. 2) in the areas with the highest overall prevalence of salmonella in cattle.

### Stability of PFGE types on farms

From 142 farms we had obtained two or more *S*. Infantis isolates during the eradication of the infection. This made it possible to follow the stability of banding patterns on the farms. The farms had various PFGE types similar to the general distribution of the types. The typing results were analysed in three groups according to the number and timely distribution of the available isolates. Data from 26 farms was analysed in more than one group as the farms had several isolates both from the same year and from different years.

From the same year, two isolates were available from 64 farms, and three or four isolates from 14 farms. The time interval between isolations varied from 0 to 9 months. On 87% (68/78) of the farms the same PFGE type was seen in at least two of the analysed isolates. Both the same type and the same subtype were seen on 50 (64%) of the 78 farms. Nine (14%) of the 64 farms with two isolates had different types. Six (43%) of the 14 farms with three or four isolates had both the same and a different type (data not shown).

From two different years, two isolates were available from 52 farms and three to five isolates from 16 farms. The time interval between isolations varied from one to three years. On 76% (52/68) of the farms the same PFGE type was seen in at least two of the analysed isolates. Both the same type and the same subtype was seen on 21 (27%) of the 68 farms. Fifteen (29%) of the 52 farms with two isolates had different types. Five (31%) of the 16 farms with three to five isolates had both the same and a different type (data not shown).

Isolates from three different years were available from 16 farms and from four different years from 6 farms. On 91% (20/22) of the farms the same PFGE type was seen in at least two of the analysed isolates. Both the same type and the same subtype was seen on 3 (14%) of the 22 farms. Nine (41%) of the 22 farms had both the same and a different type (data not shown).

The within farm differences in the *Xba*I-banding patterns of successive isolates ranged from one to five bands. Altogether 31 farms had differences. Of these farms, 32% (10/31) had a one-band difference and 55% (17/31) a two-band difference between separate isolates. For isolates from the same year, 42% (5/12) had only a one-band difference and 33% (4/12) a two-band difference, and one isolate each had a three-, four- or five-band difference. For isolates from two different years, 26% (5/19) had a one-band difference, 68% (13/19) a two-band difference and one isolate a three-band difference (data not shown).

### Ribotypes and IS*200 *types

Five combinations of ribotype and IS*200 *type were seen among the 57 analysed isolates: 1A (51 isolates), 1Q (1 isolate), 1S (1 isolate), 1T (1 isolate), and 7O (2 isolates). The IS*200 *profiles O, Q, S and T differed from profile A, which has bands of 0.6, 1.6 and 2.2 kb [7], by having either one additional band of approximately 3 kb in size (profile O), one additional band of 3.6 kb (Q), one additional band of 1.8 kb (S), or two additional bands of 3.6 and 4 kb (profile T) (data not shown). The ribotype 7 differed from type 1 by having one additional band approximately 4 kb in size (data not shown).

The most common ribo/IS*200 *type combination 1A was seen in 89% (51/57) of the isolates. The 25 analysed isolates from 1985–87 had types 1A (n = 21), 1Q (n = 1), 1S (n = 1) and 1T (n = 2). All the 19 isolates from 1992–95 had type 1A. The 13 isolates from 1999–2002 had types 1A (n = 11) and 7O (n = 2).

The 57 isolates analysed by ribotyping and IS*200*-typing, and classified into five ribo/IS*200 *type combinations, represented 13 different PFGE types. Twelve PFGE types were seen among ribo/IS*200 *type 1A isolates: type 1 (34 isolates), type 2 (6), type 35 (2), and 9 other types with one isolate each. All isolates of ribo/IS*200 *types 1Q, 1S and 7O had the same PFGE type 1. The two ribo/IS*200 *type 1T isolates included two PFGE types; of these type 153 was also seen in one isolate of ribo/IS*200 *type 1A.

## Discussion

This study describes the molecular epidemiology of an endemic bovine *S*. Infantis infection at the national level in 1985–2005, and during the eradication of the infection in individual herds. During a 35-year history of endemic Infantis infection in Finland, few other salmonella serovars have appeared in production animals in the country. Most of these serovars were seen in chicken production, and disappeared shortly. As serovar Infantis globally belongs to the most common salmonella serovars, import of new infections is possible. However, the narrowing genetic diversity of the isolates from poultry since the 1980s [7] [Pelkonen *et al*, unpublished] suggests that unrelated new Infantis infections were not imported into the main domestic infection reservoir. Restricted import of live animals and a tight control of feeding stuffs for salmonella, as well as a low level of salmonella in general, further indicate that the same Infantis infection has persisted since the first outbreak. Thus this study can be considered to analyse genetic changes occurring during an endemic infection.

The Finnish bovine *S*. Infantis isolates had a much higher prevalence of plasmids (88%) than seen in the literature [8], where plasmids were detected in only 12% of the *S*. Infantis isolates. Almost all (94%) of the more recent bovine isolates from the 1990s carried plasmids. These isolates had been stored at -70°C prior to analysis. The lower presence of plasmids (59%) in the earlier isolates may be related to their storage on egg agar slopes at room temperature [9].

PFGE analysis of isolates from 73% of all detected Infantis herds revealed that the bovine infection was highly homogeneous and clonal. Altogether 148 different *Xba*I-banding patterns including 51 PFGE types and their subtypes, were observed on 478 farms. The three most common types (1, 2 and 3) were present in 70%, 8% and 5% of the herds, respectively. The genetic relatedness (F value) of these types was within 0.90. Only seven of the remaining 48 types, containing 1% of the analysed isolates, had F values smaller than 0.70, when compared with types 1, 2 or 3.

PFGE is a rough but sensitive method for assessing genetic relatedness. Multilocus sequence typing is a more exact method, but it may not be sensitive enough to detect minor trends in clonal evolution within a salmonella serovar [10]. F values of more than 0.70 between PFGE patterns have been indicative of a clonal relationship between isolates in hospital outbreaks [11,12]. For outbreaks with long-term endemic background even a single band difference may be significant [13]. This seems to be the case with the two most common *S*. Infantis PFGE types 1 and 2, which have a one-band difference in *Xba*I restriction profile (Fig. 3). These types could also be differentiated using *Bln*I and *Spe*I [Lindqvist *et al*, unpublished], and they had different epidemiology: *Xba*I-PFGE type 1 prevailed mainly in cattle whereas the infection in broiler chickens consisted almost solely of type 2 and its subtypes [Pelkonen *et al*, unpublished].

Not many studies are published on the stability of PFGE profiles during long term salmonella infections. In the case of *Salmonella *serovar Berta, the PFGE profiles remained almost stable in strains obtained during a nationwide Danish outbreak from 1984 to 1992 [14]. For serovar Typhi, certain PFGE profiles persisted in Chile for more than 11 years [15], and two predominant PFGE profiles remained dominant from 1992 to 1999 in Papua New Guinea [16]. In our study, the predominant *S*. Infantis PFGE type 1 was detected in the 1980s as well as every year during 1992–2001. In 2002, there was only one *S*. Infantis positive farm, but in 2003, PFGE type 1 was detected again. The second most common PFGE type 2 was also seen both in the 1980s as well as annually (apart from year 1993) between 1992 and 1999. Quite interestingly, type 1 was the only type associated with the endemic bovine infection both at the start of our analysis in 1985, and, as the infection seemed to fade out, in 2003. During this period, 50 other PFGE types appeared and vanished. Thus there is no trend towards increasing diversity of PFGE profiles during these two decades. Regarding the other Finnish endemic bovine salmonella infection, caused by *S*. Typhimurium DT1, we came to the same conclusion. There certain closely related *Xba*I profiles, with identical banding patterns above 100 kb, accounted for the infections in 44% (12/27) of the herds in 1983–88 and in 68% (19/28) of the herds in 1990–99 [17].

Only a small proportion (6%) of the isolates were analysed by ribotyping and IS*200*-typing, because ribo/IS*200 *type 1A had been the only one among bovine *S*. Infantis isolates from 1988–91 [7] and 1995 [4]. Similar to the earlier studies, all isolates from 1992–95 were type 1A. The most recent bovine isolates were also mostly 1A. Three other closely related IS*200 *types were seen among older cattle isolates from the 1980s. This is in accordance with the findings from broiler chickens, in which seven closely related ribo/IS*200 *types as well as 1A were seen in 1986–91 [7]. One would assume that a change in ribotype as well as IS*200 *type would result in a change in the *Xba*I profile, too. However, types 1Q, 1S, and 7O were all classified as type 1 in PFGE. In contrast, the two strains of ribo/IS*200 *type 1T fell into different PFGE types.

The national control programme of salmonella in cattle requires bacteriological monitoring of infected herds by faecal sampling. The control restrictions that are placed upon infected herds are lifted once two negative samplings at least one month apart are obtained from the herd. Cultures received from successive samplings enabled us to follow the PFGE profiles on altogether 142 farms. Changes in *Xba*I-banding patterns were seen in 22% of the herds, but usually it was a one- or two band-difference. Three or more isolates were analysed from 52 farms, and 38% of these had two different PFGE types. The type remained the same in at least two of the analysed isolates from each farm in 87%, 76%, and 91% of the cases when the isolates were from the same year, two different years, and three or four different years, respectively.

An infection may have existed subclinically for a long time in the herd until the first salmonella isolates are obtained. At that time, a clear difference may be seen in banding patterns within the herd. If similar differences were seen between single isolates from different herds or infection sources, it would be extremely difficult to judge the significance of the finding, especially in case of an endemic infection. Analysis of several and successive isolates from each herd gives perspective for the interpretation of differences in banding patterns arising during a single infection.

In this study, neither the control restriction period nor the time of salmonella infection on the farm was recorded. However, 15% (22/142) of the farms had available salmonella isolates from a time period of three to four years. Most of the farms in this study were also included in a study [18], where the clean-up time from the first positive salmonella isolation to the second negative herd test result was studied on 227 *S*. Infantis farms in 1994–96: 20% of the herds were cleared of the infection in two months and another 28% within six months, while 44% of the herds required more than one year. In a Swedish study, the length of the restriction period varied from 22 to 1133 days (median 127 days) in 112 cattle herds infected with various salmonella serovars in 1993–2002 [19].

As salmonella infections often persist in the farm and the infective strain may undergo minor genetic changes, it would be useful to analyse several isolates from a herd both when tracing back the infection source and for descriptive epidemiology. However, there are often very few infected animals in a salmonella positive herd. Among 31 herds infected with *S*. Infantis in Finland in 1994–1995, in 16 herds only one or two animals per herd (4% of the animals) were infected. In the five most heavily infected herds, between 57% and 88% of the animals were infected [20]. As culling of infected animals is recommended for the eradication of the infection, it may be difficult to obtain several isolates from a herd during an outbreak.

After the feedborne *S*. Infantis outbreak in 1995, the 60 kb plasmid associated with the outbreak strain was found to be stable on the infected farms for up to 15 months [4]. This study indicates, that the feed-related genotype disappeared relatively rapidly afterwards. It is impossible to say, whether the particular infection faded out, or whether the genotype lost its specific plasmid. Without the 60 kb plasmid, the appearance would be not distinguishable from the dominating PFGE type 1 profile in any way. The share of farms infected with the feed-related *S*. Infantis seemed to drop steadily, from 36% of all Infantis farms in 1995 to 5% in 1998, whereafter no isolations were made but from a single farm in 2001. However, the figures are rough estimates, since isolates were available from only 30% to 60% of the *S*. Infantis infected farms in 1997–2000 (Table 1). Also, the rapid disappearance of infections caused by the feed-related strain coincides with the general decline of bovine salmonella, in particular *S*. Infantis, infections from 1997 onwards (Fig. 1).

The widely spread feedborne outbreak did not lead to a feared long-lasting salmonella problem, instead it inspired the whole cattle industry to develop and adopt effective measures to combat salmonella at the farm level. A peak in the number of non-Infantis salmonella farms in 1996, following the peak of *S*. Infantis farms in 1995 (Fig. 1), can be seen as a result of the efficient monitoring for bovine salmonella, which was performed both as a part of the national salmonella control programme and as the industry's own check-up. The rapid elimination of the *S*. Infantis infection, as well as of other bovine salmonella infections, in the late 1990s indicates that by correct control measures the infection can be eradicated or kept at an acceptable level.

## Conclusion

This study showed the value of molecular surveillance in understanding the course and control of endemic salmonella infections in an animal population. Also, it indicated that testing of several isolates from a herd in outbreak investigations is advisable, as minor changes in banding pattern between isolates from the same herd are relatively frequent during persistent infections.

## Competing interests

The authors declare that they have no competing interests.

## Authors' contributions

NL carried out the molecular genetic studies and drafted the manuscript. SP participated in the design of the study, discussed the results, and drafted and revised the manuscript. Both authors read and approved the final manuscript.
